# Causes of Death and Pathological Findings in Stranded Harbour Porpoises (*Phocoena phocoena*) from Swedish Waters

**DOI:** 10.3390/ani12030369

**Published:** 2022-02-03

**Authors:** Aleksija Neimanis, Jasmine Stavenow, Erik Olof Ågren, Emil Wikström-Lassa, Anna Maria Roos

**Affiliations:** 1 Department of Pathology and Wildlife Diseases, National Veterinary Institute, 751 89 Uppsala, Sweden; jasmine.stavenow@sva.se (J.S.); erik.agren@sva.se (E.O.Å.); emil.wikstrom@sva.se (E.W.-L.); 2 Department of Environmental Research and Monitoring, Swedish Museum of Natural History, 114 18 Stockholm, Sweden; anna.roos@nrm.se

**Keywords:** harbour porpoise, *Phocoena phocoena*, wildlife, pathology, marine mammal, health, disease, threat

## Abstract

**Simple Summary:**

Disease factors and mortality etiologies of free ranging wild cetaceans such as the harbour porpoise (*Phoceona phocoena*) are difficult to study. However, stranded animals and carcasses can provide invaluable information on the health and biology of this species. Post-mortem examinations performed on 128 stranded harbour porpoises collected over 15 years from Swedish waters examined general health, disease findings and cause of death. The main cause of death was bycatch in fishing gear (31%, confirmed or suspected). Disease, most often pneumonia, was also a frequent cause of death (21%). Porpoise population health may mirror the overall health and stability of marine ecosystems and the effects of human activities on coastal environments. Monitoring health, diseases and causes of death of porpoises allows for identification of threats to these animals, to other animals, to humans and to the environment.

**Abstract:**

Harbour porpoises (*Phocoena phocoena*) are useful indicators of the health of their wild populations and marine ecosystems, yet their elusive nature makes studying them in their natural environment challenging. Stranded porpoises provide an excellent source of data to study the health and biology of these animals and identify causes of death, diseases and other threats. The aim of this study was to document pathology, and where possible, cause of death in porpoises from Swedish waters. Post-mortem examinations were performed on 128 stranded porpoises collected from 2006 to 2020. Overall, bycatch including definitive and probable cases was the most common cause of death (31.4%), followed by disease (21.3%), predominantly pneumonia. In adults, infectious disease was the most common cause of death. Bacteria with zoonotic potential such as *Erysipelothrix rhusiopathiae* and *Brucella* sp. were documented for the first time in porpoises from Swedish waters, as was the porpoise-adapted group B *Salmonella enterica* ST416/ST417. Three of four deaths from non-infectious diseases involved parturition complications. Four cases of suspected predation were documented, but further analyses are required to confirm these findings. Our results are consistent with those from other regions in Europe and serve as a reference for future monitoring for changing patterns of health and disease of porpoises and their environments.

## 1. Introduction

Harbour porpoises (*Phocoena phocoena*) are small, coastal cetaceans that inhabit subarctic and temperate waters of the Northern hemisphere. They are the only cetacean species that resides in Swedish waters year-round. Globally, the harbour porpoise is classified as a species of least concern [1]. In Sweden, porpoises are common off the west and southwest coasts where the North Sea and Belt Sea populations reside. However, the Baltic Sea population is classified as critically endangered with an estimated 497 animals left (95% CI 80–1091) [2].

Porpoises are top predators in marine ecosystems. They have a relatively short life span compared to other top predators such as seals, dolphins and whales [3] and a comparatively more intensive reproductive cycle including earlier maturation and more frequent reproduction when compared with other odontocetes [4]. Additionally, their near-shore habitat makes them vulnerable to anthropogenic activities, which include incidental bycatch in fisheries, pollution, noise and marine traffic [5,6]. Porpoises therefore also can serve as excellent indicators of the health of our marine environments. Their aquatic habitat and shy nature make it challenging to obtain live animal data on porpoise health and biology so stranded animals provide an important source of information for this elusive species. Data from stranded porpoises in other parts of northern Europe and North America have been used to investigate cause of death, disease and other threats in these populations [6,7,8,9,10], but to our knowledge, similar data have not been published for stranded porpoises in Sweden. The purpose of this study was to collate data collected from post-mortem examinations of stranded harbour porpoises (*n* = 128) from 2006 to 2020 to provide information on cause of death, pathology and general health status for these animals. Comparison of these findings with other populations provides a baseline for future comparison to evaluate health trends of the species and their environment. We also identify knowledge gaps, potential health indicators and highlight areas for future investigation.

## 2. Materials and Methods

### 2.1. Animals

In 2008, the National Veterinary Institute, Sweden (SVA) and the Swedish Museum of Natural History (NRM) began a collaboration to perform post-mortem examinations on harbour porpoises to improve knowledge on the health, biology and threats to this species. Animals found in the field and deemed suitable for necropsy and sampling (i.e., were judged to be at most moderately decomposed, or could provide valuable samples for life history, environmental contaminant and genetics studies) were collected and stored frozen at −20 °C until necropsy sessions, which were typically held 1–2 times a year. Necropsy sessions facilitated examination of a larger number of animals even though freezing resulted in artifacts (e.g., colour change, histological artifacts) that needed to be considered during pathologic evaluation. A total of 128 stranded porpoises were examined by necropsy from 2008–2020, including three porpoises that were collected in 2006 and 2007 and stored frozen until this study began.

### 2.2. Sample and Data Collection

Standardized protocols were followed to facilitate systematic data and sample collection. Evidence of human interaction was documented using the protocol in Read and Murray [11] and necropsy examination followed Kuiken and Garcia Hartman [12]. Not all data could be collected from all animals and protocols were modified depending on the state of decomposition and/or scavenging of the carcass. 

Morphometrics were recorded and animals were weighed, photographed and examined externally for pathology, human interaction, scavenging and other abnormalities. State of decomposition was scored as mild (1), moderate (2), severe (3) or disintegrating/mummified (4), corresponding to decomposition condition codes 2–5 in Kuiken and García Hartman [12], respectively. Body condition was scored subjectively as emaciated (1; moderate to severe epaxial muscle atrophy, prominent depression dorsally between head and body, thin blubber, no internal fat), poor (2; mild epaxial muscle atrophy, i.e., mild concavity to dorsolateral silhouette when viewed from behind and no fat internally along lateral margins of the lungs), normal (3; convex dorsolateral silhouette when viewed from behind, some internal fat along lateral lung margins), and robust (4; convex dorsolateral silhouette, abundant nuchal fat and internal fat along lateral lung margins, thick blubber). Sex was recorded (male, female, undetermined) and animals were assigned to an age class (neonate, calf, juvenile or adult) based on Lockyer [13] and van Elk et al. [6]. Neonates were up to 91 cm long. Calves (young of the year assuming a July 1 birthday) included all animals with a total length >91 cm but ≤118 cm. Juvenile males were ≥118–129 cm long and juvenile females ranged from ≥118–139 cm in length. For animals that were 118 cm long, they were assigned as calves if found from April–June or juveniles if found from July–October. All males ≥130 cm long and females ≥140 cm were classified as adults.

Blubber was measured at standardized sites (dorsally, laterally and ventrally at the level of the axilla, cranial and caudal insertions of the dorsal fin and at the anus as depicted in Koopman et al. [14]) before blubber was removed to facilitate internal examination. Gross lesions were documented and when suitable, tissues were collected in 10% neutrally buffered formalin for microscopic examination. Depending on gross lesions or when needed to help determine cause of death, tissues were collected and submitted for ancillary diagnostic testing that included bacterial culture and molecular screening for viruses. Reproductive organs were measured, ovaries were assessed grossly for evidence of follicles, corpus luteum or corpora albicans and the uterus was opened and examined for the presence of a foetus. Since 2017, direct smears of testes, epididymis and the cervix were collected from animals and examined microscopically for the presence of sperm. Mammary glands were assessed for evidence of lactation. A standard set of tissues were collected for SVA’s biobank (muscle, lung, kidney, liver, spleen, brain, colon, blubber) and NRM’s environmental specimen bank for other studies including environmental contaminant analyses, and teeth, stomach contents and reproductive organs were collected for life history studies. Parasites were described, but not routinely collected throughout the study and documentation of presence or absence of a parasite in a given tissue was not consistently recorded for all animals over the entire study. For nematodes in the lungs and heart and trematodes in the liver, parasite burden was semi-quantitatively scored (none, mild, moderate, severe) for 116 and 101 porpoises, respectively.

### 2.3. Microscopic Examination

Formalin-fixed tissues were processed routinely and embedded in paraffin. Sections (3–4 µm) were stained using Mayer’s haematoxylin and eosin [15].

### 2.4. Bacteriology

All bacteriological analyses were carried out by the Department of Microbiology at SVA. Throughout the study, tissues with lesions suggestive of bacterial infection (e.g., evidence of inflammation including change in tissue colour or texture, or presence of purulent, fibrinous, necrotic or caseous material) were submitted for routine aerobic culture (*n* = 24 porpoises) and starting in October 2019, grossly normal lungs from an additional 14 porpoises with a decomposition score of moderate or less were submitted for routine aerobic culture for screening. In total, tissues from 38 porpoises were cultured. Samples were inoculated onto blood agar plates with 5% horse blood and bromocresol purple lactose agar plates and held at 37 °C under aerobic conditions. In addition, a second blood agar plate was held at 5% ± 1% CO_2_. Plates were inspected after 24 h and 48 h for bacterial growth. Any *Salmonella* sp. isolates were further characterized as described in Sandholt et al. [16]. Additionally, one porpoise had lesions suggestive of *Brucella* infection in the testis and tissue was submitted for selective *Brucella* culture. The testis sample and a positive control were inoculated in parallel onto non-selective (5% horse blood agar, bromocresol purple agar and Trypticase soy agar plates) and selective (Farell agar plates) media. Plates were held at 37 °C under aerobic conditions as well as in an 5–10% CO_2_ incubator and inspected for growth after 3 and 7 days. To further investigate this case, formalin-fixed, paraffin-embedded testis tissue was also submitted for *Brucella* species PCR. Four 6-µm-thick sections of paraffin blocks containing testis were submerged in 250 µL 0.5% Tween-20 solution. The paraffin was melted by heating the samples at 90 °C for 10 min followed by 55 °C for 5 min. The samples were centrifuged at 10,000× *g* for 15 min and then placed on ice. The hardened paraffin was removed and 195 µL of the DNA-containing Tween-solution was extracted together with 5 µL seal herpes virions using EZ1 DNA Tissue kit (Qiagen, Hilden, Germany). Real-time PCR was carried out using the PCR-assay for Brucella genus (IS711) and seal herpes (internal control) as described by Boskani et al. [17] with the following modifications; each 15 µL PCR reaction contained PerfeCTa qPCR Toughmix (Quantabio, Beverly, MA, USA), 500 nM och each primer, 100 nM of each probe and 2 µL DNA template. Real-time PCR was performed using Applied Biosystems 7500 Fast thermal cycler (Thermo Fisher Scientific Inc., Waltham, MA, USA). The PCR program comprised of an initial denaturation step of 3 min at 95 °C, followed by 45 cycles of 3 s at 95 °C and 30 s at 60 °C.

### 2.5. Virology

Animals with unknown cause of death or that had pneumonia or other bacterial infection, and had tissues in the biobank, were analysed for the presence of morbillivirus. Pooled spleen, lung and brain were submitted to the Moredun Research Institute Surveillance Unit, Scotland for morbillivirus screening by real time RT-PCR (*n* = 55) following methods described in Dagleish et al. [18]. Lung swabs from porpoises collected in 2020 with a decomposition score of <4 (*n* = 28) were analysed at the Department of Microbiology, SVA for SARS-CoV-2 using real-time RT-PCR targeting nucleoprotein, envelope and RdRp protein genes as described by Corman et al. [19].

### 2.6. Chemistry

Vaginal calculi found in the cervix of one porpoise were submitted to the Department of Chemistry, Environment and Feed Hygiene, SVA for chemical composition analysis. Analyses for environmental contaminants and biotoxins were not performed within the scope of this study.

### 2.7. Diagnoses

Animals often had more than one significant pathological finding, which resulted in multiple diagnoses for many animals. Using a system similar to Fenton et al. [10], diagnoses were categorized as primary or secondary. A primary diagnosis was defined as the diagnosis that most likely resulted in the series of events leading to stranding or death. Secondary diagnoses were defined as significant findings that may have contributed to the demise of the animal but were not deemed to be the immediate cause of death on their own. Although some degree of parasitism was a common finding, parasitic infection was only considered to be a secondary diagnosis if infections were scored as more than just mild and/or inflammatory lesions were associated with the infection. Primary diagnoses were further classified into the following categories: Bycatch, Probable bycatch, Infectious disease, Non-infectious disease (excluding emaciation), Emaciation, Trauma, Abandoned, Undetermined or Unsuitable material. ‘Bycatch’ was restricted to those animals with characteristic net marks on the head, body or extremities and froth in airways. ‘Probable bycatch’ was assigned if no net marks were evident or presence of net marks could not be assessed due to sloughed skin or scavenging, but the animal was in otherwise normal to good nutritional condition and had froth in the airways, variable levels of subcutaneous haemorrhage and had no other signs of disease or cause of death. ‘Abandoned’ was only assigned to neonates with empty gastrointestinal tracts and no other sign of disease. ‘Unsuitable material’ was assigned to carcasses where extreme decomposition and/or heavy scavenging with extensive loss of tissues precluded comprehensive examination of the carcass. These animals were often brought in for other reasons than establishing cause of death. In cases where no cause of death could be determined, the primary diagnosis was set as ‘Undetermined’. In cases where the primary diagnosis was ‘unsuitable material’ or ‘undetermined’, the animal may have had secondary diagnoses.

## 3. Results

### 3.1. Animals

**Figure 1:**
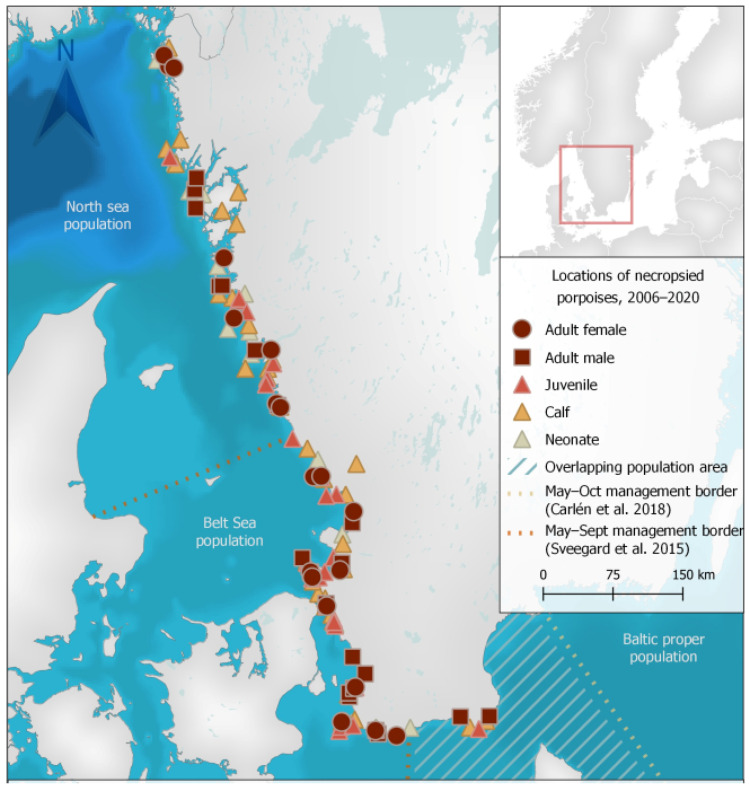
Locations of stranded harbour porpoises (*Phocoena phocoena*) collected from 2006 to 2020 in Sweden for post-mortem examination. Geodata: Carlén et al. [20], HELCOM (Open Street Map) [21], Siefert et al. [22], Sveegard et al. [23], Lantmäteriet (Sverigekartan) [24].

Stranded animals were collected and examined from the west and south-west coasts of Sweden (Figure 1). No animals were collected from the core area of the critically endangered Baltic Sea population and only six animals were collected from the overlapping area of suggested population management borders for the Belt Sea and Baltic Sea porpoise populations.

**Figure 2:**
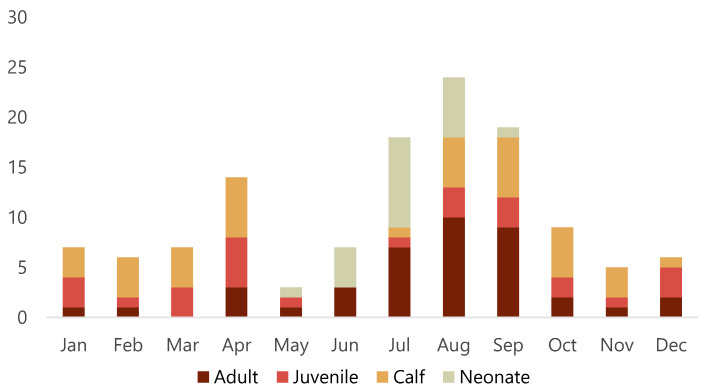
Number of harbour porpoises (*Phocoena phocoena*) that stranded per month along the Swedish coast from 2006 to 2020 and brought in for post-mortem examination.

Of the 128 porpoises examined, 64 were females, 63 were males and sex could not be determined in one animal with missing tissues. There were 21 neonates (16.4%), 40 calves (31.3%), 26 juveniles (20.3%) and 41 adults (32%). Porpoises were found during all months of the year, but there appeared to be a smaller peak of found dead animals in April and a larger peak from July to September (Figure 2). Almost two thirds (63%) of adults were found from July to September. While almost all neonates were found from June to August, two neonates were found May 24 and September 4, respectively. Detailed data for each animal is presented in Table S1.

### 3.2. Ancillary Diagnostic Analyses

#### 3.2.1. Bacteriology

Of the 38 animals from which tissues were submitted for bacterial analyses, bacteria associated with significant pathological lesions could be documented in 12 cases (Table 1). For the majority of other cases, mixed bacterial flora typical of post-mortem bacterial overgrowth were cultured, or other underlying causes for the inflammation were identified (fungal or parasitic infections).

No Brucella bacteria were cultured from the porpoise with granulomatous orchitis. However, Brucella spp. could be demonstrated by PCR on the fixed testicular tissue from this animal.

#### 3.2.2. Virology

Of the 55 porpoises tested retrospectively for morbillivirus, the sample quality of 17 was too poor for reliable analysis and results were inconclusive. No morbillivirus could be detected in the remaining animals (*n* = 38). Genetic material from SARS-CoV-2 was not detected in any of the 28 porpoises analysed.

### 3.3. Diagnoses and Causes of Death

#### 3.3.1. Primary Diagnoses

A cause of death could be determined for 88 animals (68.8%) (Table 2). In this case, 20 (15.6%) were unsuitable for post-mortem examination and no cause of death could be assigned to the remaining 20 (15.6%). The proportion of animals assigned to diagnoses ‘undetermined’ and ‘unsuitable material’ increased with increasing decomposition code (Table S1). Animals that were classified as unsuitable were excluded from further evaluation of primary diagnoses.

Excluding cases that were unsuitable for primary diagnosis, bycatch (12.0%) and probable bycatch (19.4%) when considered together were the most common primary diagnosis in our sample (31.4%), followed by undetermined cause of death (18.5%), infectious disease (17.6%), emaciation (10.2%), trauma (10.2%), abandoned (8.3%) and non-infectious disease (3.7%). Primary diagnoses according to age class are presented in Table 2. The most commonly known cause of death per age class was abandonment for neonates, bycatch or probable bycatch for calves and juveniles, and disease (infectious and non-infectious) for adults. Bycatch or probable bycatch was the next most common cause of death for adult porpoises.

Of the 13 porpoises that were clearly bycaught, 12 (92.3%) were classified as in normal to robust body condition. Animals assigned to the probable bycatch category were, by definition, in normal to robust body condition. Here, 12 of the 34 porpoises (35.3%) with a primary diagnosis of bycatch or probable bycatch had at least one secondary diagnosis, the vast majority of which was nematode parasitism in the lungs, often associated with granulomatous pneumonia.

**Figure 3:**
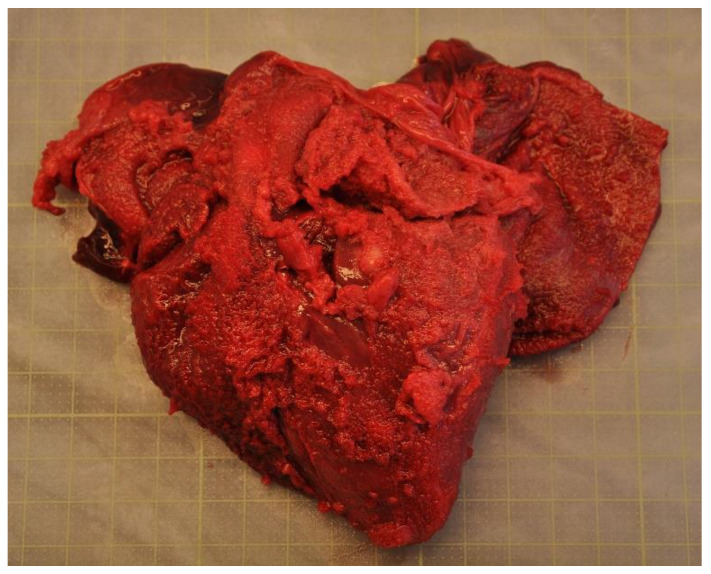
The heart from a harbour porpoise (*Phocoena phocoena*) surrounded by severe fibrinosuppurative pericarditis from *Staphylococcus aureus* infection.

**Figure 4:**
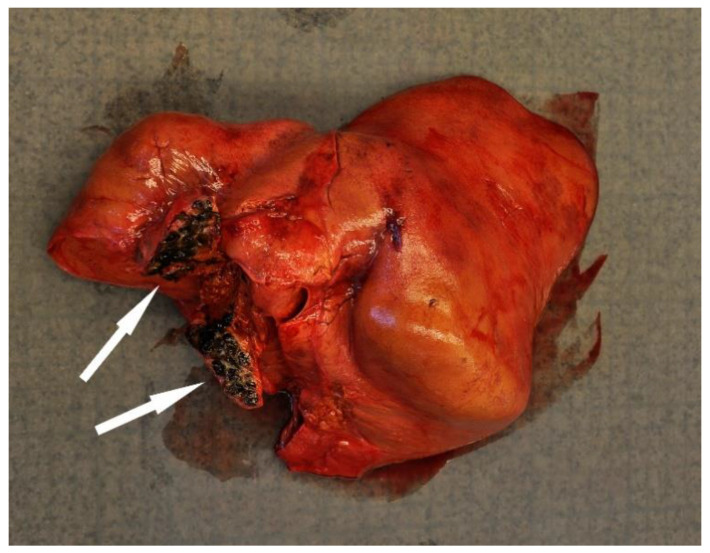
The liver from a harbour porpoise (*Phoceona phocoena*) with a locally extensive biliary trematode infection (black discoloured area shown by arrows) that obstructed bile flow. The liver is discoloured bronze-yellow and the animal had generalized icterus.

Disease was the second-most common cause of stranding or death in animals suitable for examination and of these 23 cases, infectious diseases predominated (*n* = 19). In this case, 13 (68.4%) of the 19 porpoises that succumbed to infectious disease were in poor to emaciated nutritional condition. Infectious diseases were caused by bacterial infections (*n* = 10), parasitic infections (*n* = 6), fungal infection (*n* = 2) and brain inflammation (encephalitis) of undetermined cause (*n* = 1). Of the bacterial infections, seven manifested as pneumonia. All seven of these animals also had a moderate to severe lungworm burden and severe thrombosis was evident in one of these cases. One calf suffered from sepsis as a sequela to chronic, infected bite wounds and another had a fibronosuppurative pericaditis, myocarditist and lymphadenitis (Figure 3). In the six animals diagnosed with primary parasitic infections, severe parasitic pneumonia was seen in five animals, including one animal with a large clot that obstructed airways. The sixth parasitic infection was a locally extensive, severe trematode infection morphologically consistent with *Campula oblonga* in the liver, which caused biliary obstruction, leading to liver failure and icterus (Figure 4). The fungal infections were pneumonia caused by *Aspergillus fumigatus*. Tissues from the porpoise with encephalitis were analysed for morbillivirus, but tissues were too autolysed and results were inconclusive. In total, pneumonia of various causes made up 14 of the 19 cases of infectious diseases. With respect to non-infectious disease, three of the four cases were related to parturition (one case of dystocia and two cases of stillbirth). In the fourth case, the animal suffered from severe ulcerative esophagitis and gastritis, but the underlying cause was not determined.

In cases of trauma, two had wounds consistent with ante-mortem predation (i.e., large blubber defects over the head and thorax with sharp edges accompanied by haemorrhage and regular punctures or scratches consistent with teeth or claw marks) and two other animals had wounds suspicious of predation. However, confirmatory analyses have not been performed. One post-parturient female had a ruptured uterus and another animal died from acute peritonitis following a penetrating wound into the abdomen.

#### 3.3.2. Secondary Diagnoses

**Figure 5:**
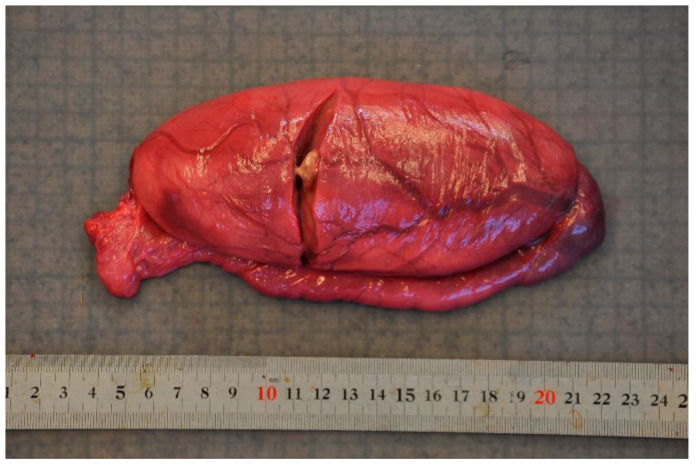
The focal, yellow, caseous abscess in the testis of an adult harbour porpoise (*Phocoena phocoena*) caused by *Brucella* sp. infection.

Secondary diagnoses (*n* = 140) were assigned in 67 animals, meaning some porpoises had more than one secondary diagnosis (Table S1). The vast majority (61%) of all secondary diagnoses were parasitic infections and associated inflammatory tissue changes. These included lungworms and associated granulomatous pneumonia (*n* = 36, including the six animals that were diagnosed with primary bacterial pneumonia), cholangitis caused by biliary trematodes morphologically consistent with *Campula oblonga* (*n* = 27), similar trematode infection of pancreatic ducts (*n* = 3), gastric ulceration caused by *Anisakis* sp. nematodes (*n* = 10) and infection of the tympanic cavity by nematodes morphologically consistent with *Stenurus minor* (*n* = 9). Other secondary diagnoses included mild to moderate inflammation of the lymphatic tissues, liver, urinary tract, central nervous system, blubber or peritoneum of unknown etiology (*n* = 15), ulceration of the gastrointestinal tract not associated with parasitism (*n* = 11), poor to emaciated nutritional condition (*n* = 9), adrenal gland cortical hyperplasia, cyst or adenoma (n = 4), hepatic lipidosis (*n* = 3), renal lipidosis (*n* = 2), struvite calculi in the cervix (*n* = 2), pox-like skin lesions (*n* = 2), granulomatous orchitis caused by *Brucella* sp. (*n* = 1) (Figure 5), congenital heart defect (*n* = 1), blunt trauma (*n* = 1), healed rib fractures (*n* = 1), pulmonary edema (*n* = 1), liver fibrosis (*n* = 1) and isolation of group B *Salmonella enterica* ST416/ST417 from the lung (*n* = 1).

#### 3.3.3. Parasitism

In this case, 70 of 116 porpoises (60%) had at least one nematode in the lungs and 36 of 101 porpoises (36%) had at least a mild infestation of trematodes in the liver morphologically consistent with *Campula oblonga*. Parasitic infection generally increased in frequency and severity in older age classes. Although 16 porpoises were recorded as having nematodes in the stomachs and 17 had nematodes in the tympanic bullae morphologically consistent with *Stenurus minor*, data was not consistently or systematically collected and recorded, precluding further interpretation of the occurrence and significance of these parasites.

## 4. Discussion

### 4.1. Animals

While stranded animals should not be considered an accurate sampling of disease incidence in the population, they provide an excellent source of data for the identification of diseases and other threats to porpoises and the potential for determining shared “One Health” factors for humans and the environment. Given the elusive nature of porpoises, stranded animals still provide access to samples for studies on the biology and population demographics of this species.

In this study, stranding locations generally reflected porpoise occurrence and densities in Swedish waters, with almost all animals originating from areas inhabited by the more abundant North Sea and Belt Sea populations (Figure 1). Given the small estimated population size of the critically endangered Baltic Sea population, it is not surprising that no dead porpoises were reported from the eastern areas of the Baltic Proper where only this population resides (Figure 1). Six porpoises in this study were collected from the overlapping area of suggested population management borders for the Belt Sea and Baltic Sea porpoise populations (Figure 1) and genetic analyses are ongoing to determine the origin of these animals. While results from this study originate primarily from animals of the North Sea and Belt Sea populations, findings are also similar to previous findings in porpoises from other parts of the Baltic Sea [25]. Our findings therefore likely give indications of the causes of morbidity and death for the Baltic Sea population, but comparisons should be carried out cautiously as habitats and conditions differ between regions. Information on the health, biology and threats to the critically endangered population in the Baltic Proper is still incomplete and targeted efforts to examine all dead animals found within their range in parallel with population genetic analyses are highly warranted. Additionally, as sample size increases, the shared morbidity and mortality factors of the North Sea and Belt Sea populations can serve as guides and where appropriate, proxies for the Baltic Sea population health concerns.

The apparent seasonal variation in stranding with more animals found in April and the summer months requires further investigation. These peaks may reflect seasonal activities (e.g., specific coastal fisheries) and vulnerable periods (e.g., peri-parturition and the first few months after birth), increased effort to detect carcasses during summer months when more people are outdoors, or likely a combination of both. Stranding data can give an estimate of frequency of species within a given region and changes in frequency of reports of dead animals can provide insights into changing population demographics or geographical shifts [26]. In future, systematic monitoring of all reported dead porpoises in Sweden is recommended because these data can complement other data sources used to monitor porpoise populations.

### 4.2. Diagnoses, Disease, Other Threats

#### 4.2.1. Bycatch and Probable Bycatch

In this study, confirmed bycatches made up 12% of the stranded animals suitable for examination. Bycatch is extremely difficult to diagnose with certainty in stranded animals and is almost surely underestimated [10]. Tell-tale net marks disappear or become obscured as skin sloughs or is removed through processes of decomposition, beaching, handling and scavenging, and not all fishing gear leave net marks. There is a delay between when animals are first reported dead and when they are collected and examined, and scavenging and autolysis proceed rapidly. To maximize the chance of detecting net marks, close coordination with field volunteers is necessary. The majority of porpoises brought in for necropsy in this study were first photographed in the field to evaluate level of decomposition. Standardized protocols for additional photographs from the field would increase the likelihood of detecting net marks.

Probable bycatch was a frequent primary diagnosis in this study. While a small proportion of animals may have been mistakenly assigned to this diagnosis class if cause of death was missed during post-mortem examination, our conservative definition of ‘probable bycatch’ likely led to the incorrect exclusion of a number of animals from this category. Recent studies of bycaught animals show that these porpoises often have concurrent morbidities and/or are in compromised nutritional condition [25,27]. Thus, even cases of probable bycatch reported here are likely an underestimate of the true number in our sample.

If bycatch and probable bycatch from human interaction are considered together, they represent the most frequent cause of death in this study. Almost one third (31.4%) of all stranded animals suitable for examination were assigned to this category. This is lower than the 38% described for stranded porpoises on English and Welsh coasts [9], but higher than the 15% described for Belgian and French coasts from 1990–2000 [8]. However, in a more comprehensive summary of causes of death of stranded porpoise deaths from 1990–2017 in Belgium, definitive bycatch made up 28% of the cases and this increased to 35% if probable bycatches were also included [28]. In other studies from German waters, bycatch was the most common cause of death of porpoises from the Baltic Sea whereas it was infrequently documented (7% of cases) in animals from the North Sea [7,29]. Although proportion of bycaught porpoises in the stranded animal sample varied between studies, bycatch accounts for a significant number of stranded animals and confirms that stranding data can be a useful indication of fishery mortality in nearshore waters. Further estimation of the magnitude of bycatch mortality requires well-designed at-sea fishery observer programs [30]. While comparison of fisheries activity (e.g., season, effort, gear used and species targeted) with porpoise strandings was beyond the scope of this study, harbour porpoises previously have been reported as bycatch in nets and trawls for a number of species such as cod (*Gadus morhua*), lumpfish (*Cyclopterus lumpus*), pollock (*Pollachius pollachius*), Atlantic mackerel (*Scomber scombrus*), spiny dogfish (*Squalus acanthias*) and langoustine (*Nephrops norvegicus*) in Swedish waters (Swedish Museum of Natural History records, unpublished data). Comparison of stranding records of cetaceans with fisheries activity will help provide insight into impacts of fisheries on porpoises in Sweden.

#### 4.2.2. Infectious Disease

Infectious disease was the next most frequent known cause of death in porpoises suitable for examination. As in other studies, pneumonia predominated infectious diseases [6,8,9]. In this study, bacterial pneumonia was always seen in association with lungworm infection. Bacteria responsible for pneumonia are consistent with reports from other European countries [6,8,31,32] and streptococcal bacteria were most common as described by Swenshon et al. [33]. Notably, a porpoise-adapted strain of *Salmonella enterica* (group B *Salmonella enterica* ST416/ST417) previously documented and definitively confirmed only in porpoises from British waters [34] was isolated for the first time in Sweden in two separate cases [16]. Host-adaptation resulted in the loss of numerous genes typically associated with increase pathogenicity, but serious, opportunistic infection was documented in one of the porpoises in this study [16]. The zoonotic potential of this Salmonella species is not known. We also document the first case of *Staphylococcus aureus* myocarditis and pericarditis in porpoises from Sweden, adding to the two cases previously described by Siebert et al. [35] in German waters. According to these authors, this bacterium is not commonly isolated from porpoises. In all three documented cases, the heart was targeted by infection, suggesting that that it may be a predilection site for *Staphylococcus aureus* infections in porpoises. The zoonotic bacteria *Erysipelothrix rhusiopathiae* has been documented in a number of marine mammal species [36] and has now also been documented on two separate occasions in porpoises from Sweden. In both cases, infection was associated with severe pneumonia. The other notable bacterial infection detected was *Brucella* sp. in the testis of a mature male harbour porpoise. Although the Brucella species was not identified, it is most probably *Brucella ceti*, a marine Brucella that infects cetaceans. This is the second documented case of orchitis in harbour porpoises caused by Brucella. Upon detection of the first case in a mature male porpoise from British waters, Dagleish et al. [37] raised the possibility of sexual transmission and concern for effects on reproductive success. Terrestrial Brucella species often cause reproductive disease and *B. ceti* has been associated with abortion in two bottlenosed dolphins (*Tursiops truncatus*) [38]. Closer monitoring of Brucella in reproductive tracts of porpoises is needed to investigate any potential impact on reproduction in porpoises in Swedish waters.

Here we report on bacterial infections deemed significant for the host because of associated inflammatory lesions. However, evaluating the significance of bacteria cultured from porpoises can be challenging. Some pathogenic bacteria have been isolated from marine mammals without causing apparent disease and a myriad of other bacteria have been cultured from porpoise tissues, but their significance is poorly understood because data on associated pathology are unavailable. Histopathological examination coupled with routine bacterial culture is recommended for all porpoises with a decomposition code of 1 or 2 to better understand the significance of bacteria isolated from porpoises.

Fungal pneumonia caused by fungi morphologically identified as *Aspergillus fumigatus* was also determined to be the cause of death in two animals. The frequency of *Aspergillus* sp. infection may be increasing in porpoises in the Netherlands [6]. If this represents a true increase, impaired immunity is hypothesized to be the reason [6] and monitoring of fungal infections in porpoises may be a useful indicator of changes in general health status. Increased surveillance of *Aspergillus* sp. infection in Swedish porpoises along with identification of environmental factors that may facilitate exposure to *Aspergillus* sp. or impact host immune response is also warranted.

As in other studies [6,7], nematodes in the airways, pulmonary vessels and the heart were a common finding in this study and heavy infections can lead to severe pneumonia which is often complicated by bacterial infection. Lungworms have also been implicated in the transmission of certain bacteria in porpoises [39]. Certain nematodes such as *Pseudalis inflexus* [6,39] are known to be particularly pathogenic for porpoises. Lungworms were not routinely identified in this study but simply assessing lungworm burdens and associated inflammation may serve as a useful indicator of porpoise health status. For example, porpoises in waters from Norway, Iceland and Greenland had milder lungworm parasitism associated with less pathology than animals from German waters, reflecting differences in host populations and/or environmental circumstances [29]. In this case, 60% of porpoises examined for presence of lungworms in this study had at least a mild burden. This is higher than the 46% reported in porpoises from German waters by Reckendorf et al. [40], but similar to prevalences of 63.8% and 69.4% of *Pseudalius inflexus* and *Torynurus convolutes*, respectively, in porpoises from the Baltic Sea population [41]. Identification and systematic recording and scoring of lungworm infection are needed to follow trends in parasitism in Swedish waters and compare findings with other porpoise populations.

The only other case where cause of death was attributed to parasitism was a locally extensive, severe biliary infection with *Campula oblonga* trematodes that blocked outflow of bile. Although 36% of porpoises examined for presence of biliary trematodes had at least a mild burden, infections were generally considered incidental findings. These findings are similar to the 42.2% prevalence reported from porpoises from British waters [42] but differ from bycaught porpoises examined in northern Norway where 90% of animals examined were parasitized with *Campula oblonga* and two thirds exhibited severe associated cholangitis and hepatitis [25].

Different viruses including morbillivirus and herpesviruses have been detected in porpoises [43,44] but, with the exception of pox-like dermatitis in two animals, no evidence of viral infection was found in this study. However, because morbilliviruses are known to impair immunity and predispose to other infections, porpoises with no known cause of death or with pneumonia or other bacterial infection were therefore screened for morbillivirus infection. No morbillivirus was detected. Following the emergence of SARS-CoV-2, cetaceans were predicted to be susceptible to infection through wastewater [45]. To rule out infection with SARS-CoV-2, porpoises that were found dead during the pandemic were screened. No evidence of infection was found.

Increased environmental contaminant burdens have been associated with immune suppression and increased risk for infectious disease in cetaceans [6,46]. Analyses of environmental contaminants in porpoises from Swedish waters, including a subset of animals from this study, are on-going to be able to assess the significance of these compounds on porpoise health.

#### 4.2.3. Non-Infectious Disease

Three of the four cases (one mature female and two neonates) with a primary diagnosis of non-infectious disease died because of birthing complications. Neonates made up a relatively large proportion (16.4%) of the animals examined in this study and reproductive complications may have contributed to the deaths of neonates assigned to other primary diagnosis categories. Similar reproductive complications are not uncommon in porpoises from British waters and environmental contaminants may play a role [47]. Further investigation and monitoring of reproductive status and failure in porpoises in Sweden would serve as a useful health indicator for these populations.

Non-infectious diseases also comprised a number of the secondary diagnoses. Gastrointestinal ulceration was the most common and some cases were likely manifestations of stress.

No pathological evidence of intoxication was seen but analyses for biotoxins were not carried out in this study. It is possible that cases of acute toxicosis from biotoxin exposure were missed. However, all porpoises examined in this study were from single stranding events and there was no history of concurrent mortality events in other species, for example fish or seabirds. Samples for biotoxin analyses are now collected and archived routinely, and future evaluation of biotoxin exposure in porpoises from Swedish waters is highly warranted.

#### 4.2.4. Trauma

Predator-related trauma was suspected to be the cause of death in four porpoises in this study. Grey seals (*Halichoerus grypus*) have emerged as significant predators of harbour porpoises in the North Sea [48,49] and grey seals and porpoise range overlap in Swedish waters, particularly in the Baltic Sea. Lesions observed in these four cases are consistent with those described for grey seal attacks, but other predators to consider include the group of killer whales (*Orcinus orca*) now regularly sighted off the Swedish west coast and sharks. Predation from other marine species has not been documented in Sweden and, if confirmed through genetic analyses (environmental DNA analyses of wounds), may represent a new threat to porpoises.

#### 4.2.5. Undetermined or Unsuitable Material

The proportion of animals with a primary diagnosis of ‘undetermined’ or ‘unsuitable material’ increased as degree of decomposition increased, reflecting that fact that comprehensive necropsy examination is precluded by advanced autolysis. Despite this, a primary diagnosis could still be assigned in just over half of the animals that were severely autolyzed (decomposition code of 3). Severely autolyzed animals (especially adults and those potentially originating from endangered populations like the Baltic Sea) provide important samples and data for other studies (e.g., life history and population genetics). Based on our findings, there is some value in also performing necropsy examinations on these animals despite the limitations associated with advanced autolysis.

## 5. Conclusions

This study provides an important first description of causes of death, diseases, pathology and potential population threats in porpoises inhabiting Swedish waters and serves as a reference for future monitoring of trends. Findings presented here are generally consistent with findings from other regions in the North Atlantic and a number of diseases were documented for the first time in porpoises from Sweden.

Bycatch and probable bycatch when considered together was the most common cause of death, confirming that fishery interactions are still a common threat to porpoises in Swedish waters. Infectious disease was also common, particularly in adult animals. Animals with impaired immunity are more susceptible to infections. Impaired immunity in porpoises may be associated with viral infections, malnutrition or environmental contaminants [6]. We found no evidence of underlying viral infections in our study. Although two thirds of animals with a primary diagnoses of infectious disease were in poor or emaciated nutritional condition, it is difficult to assess whether compromised nutritional status led to impaired immunity and disease, or whether it was the result of disease. Environmental contaminant burdens were not investigated in this study. Given that reproductive failure may also be related to environmental contaminants, comparison of health and reproductive data with environmental contaminant burdens in Swedish porpoises will help fill the knowledge gaps on the effects of contaminants on harbour porpoises in Swedish waters.

Stranded animals in this study represent a combination of ill or debilitated animals (e.g., those with disease or that are emaciated) and animals more representative of the general population (e.g., those that died from acute trauma or in fishery interactions but are otherwise without significant disease). In order to better understand the health and biology of harbour porpoises, both types of animals are needed. Sick and debilitated animals provide information on pathogens and other disease conditions of porpoises and the threats that they are exposed to whereas animals more representative of the general population are needed to follow trends in body condition, growth, reproduction, dietary habits and environmental contaminants. Since porpoise health mirrors health of the ecosystem in which they live, changing patterns of health and disease often signal ecosystem change. Long-term monitoring is needed to detect these changes.

## Figures and Tables

**Table 1:** Bacterial infections of stranded harbour porpoises (*Phocoena phocoena*) examined form Swedish waters from 2006 to 2020.

Identification Number	Sex	Age Class	Bacteria Cultured	Tissue(s) Cultured	Lesion
12-VLT000154	M	Calf	*Staphylococcus aureus*	Pericardium, lymph node	Myocarditis, pericarditis, lymphadenitis
16-VLT001592	M	Adult	*Brucella* sp. *	Testis	Orchitis
17-VLT002645	F	Adult	*Edwardsiella tarda*	Heart valve	Endocarditis and pulmonary thrombosis
17-VLT002646	F	Adult	*Streptococcus canis*		Sepsis
17-VLT002652	M	Adult	group B *Salmonella enterica* ST416/ST417	Lung	Pneumonia
18-VLT001113	M	Adult	*Schwanella putrefaciens*	Lung	Pneumonia
19-VLT002835	F	Juvenile	*Streptococcus* sp.	Lung	Pneumonia
19-VLT002839	F	Adult	*Streptococcus* sp.	Lung	Pneumonia
19-VLT002851	M	Calf	*Erysipelothrix rhusiopathiae*	Lung	Pneumonia
20-VLT001389	F	Juvenile	group B *Salmonella enterica* ST416/ST417	Lung	Pneumonia **
20-VLT001392	M	Calf	*Streptococcus phocae*	Dermal abscess, lung, spleen	Sepsis
20-VLT002367	M	Adult	*Erysipelothrix rhusiopathiae*	Lung	Pneumonia

* No growth on selective *Brucella* culture but detected through molecular analysis (PCR)*;* ** Detected through routine culture but the associated pneumonia was attributed to the concurrent lungworm infection.

**Table 2:** Causes of death of stranded harbour porpoises (*Phocoena phocoena*) collected from Swedish waters from 2006 to 2020.

Cause of Death	Neonate	Calf	Juvenile	Adult	Total
Bycatch	0	7	4	2	13
Probable bycatch	1	7	5	8	21
Infectious disease	0	6	3	10	19
Non-infectious disease	2	0	0	2	4
Trauma	0	5	3	3	11
Emaciation	3	4	1	3	11
Abandoned	9	0	0	0	9
Undetermined	2	6	5	7	20
Unsuitable	4	5	5	6	20

## Data Availability

All data presented in this study are available in Table S1.

## Supplementary Materials

The following is available online at https://www.mdpi.com/article/10.3390/ani12030369/s1, Table S1: Metadata for 128 stranded harbour porpoises (*Phocoena phocoena*) that stranded in Sweden from 2006 to 2020 and were examined by necropsy.
