# Supplementary material for: Causes of Death and Pathological Findings in Stranded Harbour Porpoises (Phocoena phocoena) from Swedish Waters
Source: Animals (Basel). 2022 Feb 3;12(3):369. doi: 10.3390/ani12030369 (PMC8833703; doi:10.3390/ani12030369)
Supplement: Supplementary file 1 [file animals-12-00369-s001.zip › animals-1555842-supplementary.pdf]

Table S1. Metadata for 128 stranded harbour porpoises (*Phocoena phocoena*) that stranded in Sweden from 2006 to 2020 and were examined by necropsy.

SVA ID	NRM ID	Date Found	Location	Coordinate N (RT90)	Coordinate E (RT90)	Sex	Age class	Length (cm)	Nutritional status [a]	Decomposition Score [b]	Primary Diagnosis category	Primary diagnosis	Secondary diagnosis(es)
09-VLT000508	C2007/08061	2007-12-14	Onsala	6400365	1263474	F	Juvenile	125,5	3	1	Probable bycatch	Probable bycatch	Moderate granulomatous pneumonia associated with moderate lungworm infection; mild granulomatous steatitis
09-VLT000509	C2008/08899	2008-12-21	Bo	6492877	1235776	F	Calf	113,5	3	3	Infectious disease	Fungal pneumonia (*Aspergillus sp.*)	Mild cholangitis
09-VLT000722	C2007/08062	2007-10-16	Malöviken	6384800	1268222	F	Calf	100,5	1	3	Emaciation	Emaciation	
09-VLT000723	C2007/08063	2006-09-25	Lysekil	6470736	1243532	M	Calf	109,5	3	2	Probable bycatch	Probable bycatch	
09-VLT001657	C2009/02664	2009-06-01	Kungälv	6419774	1252395	M	Neonate	69,0	1	1	Emaciation	Emaciation	
09-VLT001659	C2009/02666	2009-04-01	Strömstad	6544445	1232847	M	Calf	115,0	3	2	Trauma	Blunt trauma	
09-VLT001660	C2009/04448	2009-06-08	Vrångö	6389340	1259900	F	Adult	159,5	3	1	Non-infectious disease	Dystocia	Mild gastric ulceration with associated Anisakis infection; moderate biliary trematode infection
09-VLT001661	C2009/04450	2009-08-25	Ljunghusen	6146021	1317856	F	Neonate	70,5	3	2	Undetermined	Undetermined; head missing	
09-VLT001662	C2009/04451	2009-08-26	Ljunghusen	6144384	1318573	F	Juvenile	119,0	.	3	Unsuitable material	Unsuitable material; severely decomposed	

09-VLT001884	C2009/07366	2009-09-17	Ribersberg	6167647	1320986	F	Adult	.	3	3	Undetermined	Undetermined	Mild to moderate granulomatous pneumonia associated with moderate lungworm infestation; moderate Anisakis-associated ulceration in forestomach; mild to moderate biliary trematode infection with associated cholangitis; moderate nematode infection in tympanic cavity
10-VLT001480	C2010/05198	2010-08-17	Båstad	6259600	1317000	F	Neonate	78,0	3	4	Non-infectious disease	Stillborn	
10-VLT002074	C2010/05196	2010-09-24	Helsingborg	6217000	1306000	M	Calf	104,5	3	2	Probable bycatch	Probable bycatch	
10-VLT002075	C2010/05197	2010-07-20	Helsingborg	6217000	1306000	M	Neonate	89,5	3	1	Probable bycatch	Probable bycatch	
10-VLT002545	C2010/10094	2010-07-22	Älvsborgsbron	6403000	1267000	F	Neonate	83,0	2	3	Abandoned	Abandoned	Emaciated
10-VLT002546	C2010/10096	2010-08-02	Halland	6294475	1301439	F	Adult	156,0	2	2	Bycatch	Bycatch	Mild granulomatous pneumonia associated with moderate

													lungworm infection
10-VLT002547	C2010/10098	2010-10-31	Varberg	6336000	1284000	F	Adult	144,0	.	2	Undetermined	Undetermined	Moderate to severe lungworm infection associated with moderate granulomatous pneumonia; mild to moderate biliary trematode infection with associated cholangitis; moderate to severe nematode infection in tympanic cavity
10-VLT002548	C2010/10095	2010-09-02	Saltholmen	6399000	1263000	M	Juvenile	128,0	1	3	Emaciation	Emaciation	Mild encephalitis; moderate lungworm infection; mild focal gastric ulceration in forestomach
10-VLT002549	C2010/10097	2010-10-19	Ringhals	6353100	1277142	F	Juvenile	118,0	3	1	Undetermined	Undetermined	
12-VLT000150	C2012/00005	2010-11-16	Ringhals	6354142	1277286	M	Calf	96,5	4	1	Trauma	Blunt trauma	
12-VLT000151	C2012/00007	2011-04-16	Höganäs	6238000	1297000	M	Adult	150,0	3	3	Probable bycatch	Probable bycatch	
12-VLT000152	C2012/00009	2011-05-31	Gottskär	6370000	1271000	M	Adult	154,5	3	1	Probable bycatch	Probable bycatch	Mild granulomatous pneumonia

													associated with moderate lungworm infection; moderate gastric ulceration associated with moderate Anisakis parasitism; mild steatitis; adrenal cyst; mild to moderate biliary and pancreatic duct trematode infection
12-VLT000153	C2012/00006	2011-04-16	Höganäs	6238000	1297000	F	Adult	145,0	2	1	Probable bycatch	Probable bycatch	Mild to moderate granulomatous pneumonia associated with moderate to severe lungworm infection
12-VLT000154	C2012/00008	2011-08-15	Vändelsö	6359000	1278000	M	Calf	103,0	2	1	Infectious disease	Bacterial pericarditis and myocarditis (*Staphylococcus aureus*), sepsis	Mild to moderate lungworm infection
12-VLT000155	C2012/00004	2011-08-17	Trelleborg	6137000	1345000	F	Adult	169,0	1	4	Emaciation	Emaciation	
12-VLT000156	C2012/00001	2011-05-27	Falsterbo	6144000	1311000	F	Juvenile	127,5	3	3	Unsuitable material	Unsuitable material; heavily decomposed and scavenged	

12-VLT000157	C2012/00003	2011-09-05	Barsebäck kommun	6186000	1320000	M	Adult	155,0	2	3	Unsuitable material	Unsuitable material; severely decomposed	
12-VLT000158	C2012/00002	2011-12-07	Bunkeflo strand	6162000	1317000	M	Adult	136,0	3	2	Probable bycatch	Probable bycatch	
12-VLT002016	C2012/01697	2012-xx-xx	Råå strand	6212033	1308423	F	Calf	105,5	3	1	Probable bycatch	Probable bycatch	Granulomatous splenitis and lymphadenitis
12-VLT002017	C2012/01696	2012-03-22	Höganäs	6232411	1298055	M	Calf	118,0	4	1	Bycatch	Bycatch	
12-VLT002018	C2012/01698	2012-07-25	Alnarp	6212033	1308423	M	Neonate	85,0	1	3	Emaciation	Emaciation	Blunt trauma
12-VLT002019	C2012/01695	2012-xx-xx	Skälderviken	6242520	1315912	M	Adult	143,5	2	3	Undetermined	Undetermined	Moderate granulomatous pneumonia associated with moderate lungworm infection; poor condition; mild to moderate biliary and pancreatic duct trematode infection; mild to moderate nematode infection in tympanic cavity
13-VLT003451	C2013/07074	2013-04-12	Helsingborg	6222000	1305000	M	Calf	118,0	3	2	Bycatch	Bycatch	
13-VLT003452	C2013/07070	2012-08-20	Göteborg, Torslanda	6401090	1259700	M	Calf	97,5	1	1	Emaciation	Emaciation	
13-VLT003453	C2013/07072	2013-10-06	Lappesand	6403589	1252063	M	Calf	.	3	3	Undetermined	Undetermined	
13-VLT003455	C2013/07069	2012-11-18	Halmstad	6283000	1320000	F	Calf	109,0	1	3	Infectious disease	Parasitic pneumonia	Emaciation; vagoliths (struvite)

13-VLT003456	C2013/07067	2012-04-01	Mölle	6253000	1297000	F	Juvenile	121,0	3	2	Bycatch	Bycatch	
13-VLT003457	C2013/07075	2012-03-30	Ängelholm	6239000	1317000	F	Calf	101,5	3	3	Bycatch	Bycatch	
13-VLT003458	C2013/07068	2012-04-06	Rågelund	6361800	1281200	F	Juvenile	127,0	3	2	Bycatch	Bycatch	
13-VLT003459	C2013/07073	2013-09-04	Helsingborg	6207003	1309744	F	Neonate	84,0	1	2	Emaciation	Emaciation	Healed rib fractures
15-VLT001999	C2015/05286	2015-02-23	Myggstadsviken	6424192	1256195	F	Adult	162,5	2	1	Non-infectious disease	Ulcerative esophagitis and gastritis	Vagoliths, moderate biliary trematode infestation with chronic locally extensive cholangitis; mild to moderate lungworm infection
15-VLT002000	C2014/09319	2014-08-23	Getterön	6338384	1281819	F	Adult	155,0	3	3	Undetermined	Undetermined	Mild to moderate lungworm infection, mild biliary trematode infection with mild associated cholangitis
15-VLT002001	C2015/05285	2015-01-13	Lysekil	6470583	1243426	M	Adult	142,0	3	1	Bycatch	Bycatch	Adrenal adenoma; moderate biliary trematode infestation with associated cholangitis

15-VLT002002	C2015/05643	2015-08-03	Trelleborg	6142600	1332600	F	Neonate	89,5	3	3	Unsuitable material	Unsuitable material	
15-VLT002003	C2015/05288	2015-04-28	Tollenäs	6443138	1264534	M	Calf	113,0	1	1	Emaciation	Emaciation	Esophageal ulceration
15-VLT002004	C2015/05287	2015-04-18	Askön	6392523	1267350	F	Juvenile	132,0	3	3	Unsuitable material	Unsuitable material	Mild to moderate lungworm infection; mild Anisakis infection in forestomach with associated ulcer
15-VLT002044	C2015/05597	2015-09-29	Skanörhamn	6147020	1311680	F	Calf	109,0	2	3	Unsuitable material	Unsuitable material	Poor nutritional condition
15-VLT002045	C2015/05598	2015-09-17	Möllöviken	6147960	1319780	M	Calf	98,0	3	4	Unsuitable material	Unsuitable material	
16-VLT001260	A2016/05539	2016-07-26	Laholm	6272842	1324285	F	Adult	165,0	1	2	Infectious disease	Parasitic pneumonia with intrabronchial thrombus	Gastric ulcer with severe Anisakis infestation; emaciation; dermatitis
16-VLT001589	A2016/05526	2016-09-06	Helsingborg	6164028	1288957	M	Juvenile	124,0	3	3	Undetermined	Undetermined	
16-VLT001590	A2016/05522	2016-02-01	Pinnevik	6457585	1232271	M	Calf	117,0	2	2	Infectious disease	Parasitic pneumonia	Poor nutritional condition
16-VLT001592	A2016/05524	2016-07-16	Munkevik	6464000	1242000	M	Adult	143,0	3	2	Probable bycatch	Probable bycatch	Granulomatous orchitis (*Brucella sp.*); moderate biliary trematode infection with moderate associated cholangitis

16-VLT001594	A2016/05527	2015-12-11	kämpersvik	6483299	1229242	M	Juvenile	119,5	3	1	Trauma	Traumatic peritonitis	
16-VLT001595	A2016/05521	2016-03-17	Smögen	6463824	1225788	F	Calf	103,5	3	2	Probable bycatch	Probable bycatch	
16-VLT001596	A2016/05523	2016-07-14	Onsala	6352555	1244550	M	Neonate	80,0	1	4	Unsuitable material	Unsuitable; severely decomposed	
16-VLT001622	A2016/05528	2016-xx-xx	Bropelarna till Kph	6163400	1316200	F	Calf	114,0	4	1	Unsuitable material	Unsuitable; scavenged and internal organs missing	
16-VLT002068	A2016/05637	2016-10-27	Helsingborg	6218828	1305391	M	Adult	141,5	3	1	Infectious disease	Parasitic biliary obstruction (cholestasis) leading to severe hepatic necrosis and icterus	Moderate to severe gastric ulceration (pylorus); moderate Anisakis infection; chronic colitis
17-VLT001479	A2017/05218	2017-04-04	Höganäs	6234900	1297800	U	Juvenile	123,0	3	3	Unsuitable material	Unsuitable material; severely decomposed and scavenged	
17-VLT001480	A2017/05219	2017-04-09	Mölle hamn	6244300	1294800	M	Calf	118,0	3	2	Undetermined	Undetermined	Focal chronic cholangitis (likely parasite-related)
17-VLT001481	A2017/05216	2017-03-16	Skrea strand	6311200	1298600	M	Calf	112,0	4	1	Bycatch	Bycatch	
17-VLT001482	A2017/05215	2017-01-11	Halmstad	6282813	1308449	F	Juvenile	128,0	3	1	Probable bycatch	Probable bycatch	Severe lungworm infection with associated pneumonia
17-VLT001483	A2017/05217	2017-03-22	Höganäs	6234795	1301777	F	Juvenile	129,5	2	2	Infectious disease	Parasitic pneumonia	Mild to moderate biliary trematode infection with associated

													cholangitis; moderate Anisakis infection with associated ulceration in forestomach; poor nutritional condition
17-VLT001484	A2017/05214	2016-11-30	Helsingborg	6237728	1305364	F	Juvenile	133,0	3	1	Probable bycatch	Probable bycatch	Mild to moderate Anisakis infection of forestomach with associated ulceration; mild to moderate lungworm infection
17-VLT001485	A2017/05220	2017-04-22	Skälderviken	6253937	1317018	M	Calf	116,5	4	1	Probable bycatch	Probable bycatch	
17-VLT002644	A2017/05594	2017-06-26	Helsingborg	6217300	1305700	F	Adult	162,0	2	1	Probable bycatch	Probable bycatch	Pox-like dermatitis
17-VLT002645	A2017/05600	2017-08-13	Kungsbacka	6369737	1280315	F	Adult	161,5	2	2	Infectious disease	Pulmonary thrombus and bacterial endocarditis (*Edwardsiella tarda*)	Bacterial and parasitic pneumonia associated with moderate lungworm infection; moderate to severe gastric ulceration (forestomach); moderate biliary trematode

													infection with moderate cholangitis
17-VLT002646	A2017/05598	2017-08-04	Särdal	6294500	1306000	F	Adult	162,0	2	1	Infectious disease	Bacterial sepsis and meningoencephalitis (*Streptococcus canis*)	Moderate lungworm infection associated with mild to moderate pneumonia, adrenal adenoma; moderate to severe esophageal and forestomach ulceration; moderate biliary and pancreatic duct trematodes with associated ductal fibrosis and inflammation
17-VLT002647	A2017/05602	2017-09-17	Hälsö	6374780	1250849	M	Adult	139,5	1	1	Emaciation	Emaciation	
17-VLT002648	A2017/05601	2017-08-24	Mölle, Höganäs	6246611	1292457	M	Adult	142,0	2	3	Undetermined	Undetermined	Mild to moderate cholangitis associated with moderate biliary trematode infection; mild mononuclear radiculitis; mild to moderate lungworm

													infection; mild ulceration in forestomach
17-VLT002649	A2017/05593	2017-06-19	Hällevik, Orust	6453520	1241855	M	Adult	144,0	3	1	Infectious disease	Encephalitis	Mild to moderate granulomatous pneumonia associated with mild to moderate lungworm infection; mild to moderate parasite-induced cholangitis
17-VLT002650	A2017/05597	2017-07-19	Sunnäsfjorden	6488007	1227584	M	Calf	109,5	2	1	Emaciation	Emaciation	
17-VLT002651	A2017/05599	2017-08-13	Särdal	6294000	1306000	M	Juvenile	122,5	2	2	Undetermined	Undetermined	Mild to moderate lungworm infection associated with mild pneumonia; mild esophageal ulceration
17-VLT002652	A2017/05596	2017-07-11	Getterön	6337258	1282549	M	Adult	139,5	1	1	Infectious disease	Bacterial pneumonia (group B *Salmonella enterica* ST416/ST417)	Moderate to severe lungworm infection; mild to moderate biliary trematode infection with associated cholangitis

17-VLT002678	A2017/05595	2017-07-03	Skanör	6147000	1312000	F	Adult	147,0	2	1	Trauma	Acute blunt trauma	
18-VLT001111	A2018/05286	2018-04-08	Höganäs	6234283	1297751	M	Juvenile	122,0	3	1	Probable bycatch	Probable bycatch	
18-VLT001112	A2018/05287	2018-04-14	Orust	6461101	1266616	F	Calf	105,5	3	3	Unsuitable material	Unsuitable material; severe decomposition	
18-VLT001113	A2018/05283	2017-11-20	Lomma	6164336	1316931	M	Adult	159,5	3	2	Infectious disease	Bacterial pneumonia (*Schwanella putrefasciens*)	Moderate lungworm infection with associated pneumonia; mild to moderate biliary trematode infection with associated cholangitis; pox-like dermatitis; mild steatitis
18-VLT001114	A2018/05284	2017-12-18	Mellby strand	6266129	1323113	M	Adult	150,0	2	2	Undetermined	Undetermined	Mild to moderate lungworm infection
18-VLT002397	A2018/05707	2018-06-28	Helsingborg	6219068	1305362	F	Neonate	78,0	2	3	Non-infectious disease	Stillborn	
18-VLT002398	A2018/05713	2018-10-13	Oskarström	6300910	1327089	M	Calf	109,0	4	1	Bycatch	Bycatch	
18-VLT002399	A2018/05712	2018-09-05	Trelleborg	6138695	1333695	M	Adult	132,5	3	3	Trauma	Trauma	
18-VLT002400	A2018/05708	2018-07-09	Sydkoster	6538131	1225841	F	Neonate	82,0	3	2	Abandoned	Abandoned	Gastric hemorrhage
19-VLT000831	A2019/05291	2019-01-07	Ringhals	6354100	1277300	M	Calf	111,5	3	1	Trauma	Trauma	Mild to moderate lung edema; mild to moderate

													lungworm infection with focal, mild associated pneumonia; mild biliary fluke infestation with mild, focal cholangitis
19-VLT000832	A2019/05293	2019-02-14	Domsten	6225000	1301000	M	Calf	115,0	4	3	Undetermined	Undetermined; predation or post-mortem scavenging of head	
19-VLT000833	A2019/05294	2019-02-26	Varberg	6336500	1283900	F	Calf	104,0	4	3	Undetermined	Undetermined, wounds suggestive of predation	
19-VLT000834	A2019/05292	2019-01-30	Rydebäck	6208000	1309000	M	Calf	110,0	4	1	Trauma	Trauma, corkscrew lesions	Mild lungworm infection with mild to moderate associated pneumonia
19-VLT000835	A2019/05295	2019-03-11	Vejbystrand	6246000	1311000	M	Juvenile	121,0	4	1	Trauma	Trauma, anthropogenic or predation	
19-VLT002835	A2019/05578	2019-08-16	Helsingborg	6209069	1309680	F	Juvenile	132,5	3	1	Infectious disease	Parasitic and bacterial pneumonia (*Streptococcus* sp.)	Mild, focal biliary fluke infestation with mild, focal cholangitis
19-VLT002836	A2019/05585	2019-09-29	Lomma	6175464	1327253	M	Adult	144,5	2	2	Undetermined	Undetermined	Chronic stomach ulceration associated with severe Anisakis infection; moderate biliary

													trematode infection with associated cholangitis; mild to moderate lungworm infection
19-VLT002839	A2019/05576	2019-07-14	Rossö, Buhuslän	6534072	1232107	F	Adult	155,0	2	3	Infectious disease	Parasitic and bacterial pneumonia (*Streptococcus* sp.)	Subacute peritonitis; ulcerative gastritis associated with moderate Anisakis infection; poor condition; adrenocortical hyperplasia; moderate biliary trematode infection
19-VLT002840	A2019/05582	2019-08-26	Norra Björkö	6408317	1254287	M	Adult	138,0	2	3	Unsuitable material	Unsuitable; severely decomposed	Moderate lungworm and biliary trematode infection
19-VLT002843	A2019/05574	2019-06-10	Särö, Stallviken	6381069	1268407	F	Neonate	80,0	3	2	Abandoned	Abandoned	Poor nutritional condition
19-VLT002844	A2019/05584	2019-09-13	Trelleborg	6140785	1332451	M	Adult	144,0	3	3	Unsuitable material	Unsuitable; severely decomposed	Severe lungworm infection in airways and vessels; mild to moderate biliary

													trematode infection
19-VLT002848	A2019/05580	2019-08-21	Ålabodarna	6206395	1310487	F	Neonate	86,0	3	1	Undetermined	Undetermined (probable drowning-bycatch or predation)	
19-VLT002851	A2019/05581	2019-08-26	Haverdalsstrand	6292817	1307518	M	Calf	114,5	2	3	Infectious disease	Bacterial pneumonia (*Erysipelothrix rhusiopathiae*)	Moderate lungworm infection of vessels and airways
19-VLT002854	A2019/05577	2019-08-06	Risteören	6147367	1402178	M	Adult	140,0	2	3	Infectious disease	Parasitic pneumonia	Poor body condition; mild to moderate biliary trematode infection
19-VLT002855	A2019/05579	2019-08-11	Bingsmarken	6141743	1353541	M	Neonate	83,5	3	4	Unsuitable material	Unsuitable; severely decomposed	
19-VLT002856	A2019/05583	2019-09-01	Sandbystrand	6144599	1400138	F	Calf	105,0	3	4	Trauma	Trauma consistent with predation	
19-VLT002857	A2019/05586	2019-10-10	Nidingen	6359754	1264727	M	Calf	103,5	3	2	Unsuitable material	Unsuitable material; severely decomposed and scavenged	
20-VLT001389	A2020/05381	2020-02-08	Helsingborg	6217280	1305760	F	Juvenile	121,5	4	1	Bycatch	Bycatch	Moderate nematode infection in lungs and tympanic cavity; group B *Salmonella enterica* ST416/ST417 cultured from lung
20-VLT001390	A2020/05378	2020-01-12	Onsala	6349872	1276219	F	Juvenile	118,5	4	1	Trauma	Trauma consistent with predation	

20-VLT001391	A2020/05374	2019-07-27	Stenungsund	6461081	1246391	M	Neonate	85,0	3	3	Abandoned	Abandoned	
20-VLT001392	A2020/05377	2020-01-10	Svanvik	6451244	1256708	M	Calf	105,5 +/- 2	2	1	Infectious disease	Bacterial infection (sepsis, *Streptococcus phocae*); old infected bite wounds	
20-VLT001393	A2019/05575	2019-07-11	Malmö	6167672	1321013	F	Neonate	88,0	3	4	Unsuitable material	Unsuitable material	
20-VLT001394	A2020/05375	2019-11-13	Nidingen	6347402	1242143	F	Calf	110,5	4	1	Undetermined	Undetermined	Oral cavity infection
20-VLT001395	A2020/05385	2020-05-27	Strömsund	6480161	1231702	M	Neonate	81,0	3	1	Abandoned	Euthanised (abandoned)	
20-VLT001397	A2020/05379	2020-01-31	Helsingborg	6207562	1309674	F	Juvenile	125,5	3	1	Bycatch	Bycatch	Severe lung parasitism with associated granulomatous pneumonia; severe nematode infection in tympanic cavity
20-VLT001398	A2020/05382	2020-03-18	Lerhamn	6241493	1296419	F	Juvenile	124,0	4	1	Undetermined	Undetermined	Mild to moderate nematode infection of tympanic cavity
20-VLT001399	A2020/05380	2020-02-05	Helsingborg	6217167	1305208	M	Calf	118,0	4	1	Bycatch	Bycatch	
20-VLT001400	A2020/05383	2020-04-08	Strömstad	6540384	1229822	F	Adult	148,0	3	1	Probable bycatch	Probable bycatch	Severe lungworm infection with associated granulomatous pneumonia
20-VLT001401	A2020/05376	2019-12-26	Skanör	6141422	1310042	M	Juvenile	118,5	2	1	Infectious disease	Fungal infection (pneumonia, *Aspergillus* sp.);	Congenital heart defect; dermatitis;

													mild to moderate lungworm infection
20-VLT002012	A2020/05728	2020-08-24	Höganäs	6234950	1297830	F	Adult	158,7	1	3	Emaciation	Emaciation	
20-VLT002255	A2020/05731	2020-08-29	Kåseberga	6140875	1390047	F	Calf	107,7	4	3	Probable bycatch	Probable bycatch	
20-VLT002256	A2020/05730	2020-08-25	Trelleborg	6140757	1332198	F	Adult	142,3	3	4	Unsuitable material	Unsuitable material	
20-VLT002365	A2020/05732	2020-09-09	Ystad	6123601	1392771	F	Juvenile	131,0	3	4	Unsuitable material	Unsuitable material	Mild to moderate lungworm infection
20-VLT002366	A2020/05735	2020-09-17	Helsingborg	6218793	1305362	F	Calf	111,0	4	3	Bycatch	Bycatch	
20-VLT002367	A2020/05734	2020-09-16	Naturum Öresund	6140362	1294171	M	Adult	132,0	3	3	Infectious disease	Bacterial (*Erysipelothrix rhusiopathiae*) and parasitic pneumonia	Gastric ulcers
20-VLT002369	A2020/05733	2020-09-12	Ystad	6147395	1384409	M	Adult	145,0	3	4	Unsuitable material	Unsuitable material	Mild to moderate lungworms, biliary trematodes and tympanic cavity nematode infection
20-VLT002422	A2020/05737	2020-09-21	Skanör	6146822	1311672	F	Adult	157,6	2	3	Unsuitable material	Unsuitable material	Mild to moderate granulomatous pneumonia with moderate lung worms; mild to moderate nematodes in tympanic cavity

20-VLT002959	A2020/05736	2020-10-13	Helsingborg	6217189	1304741	F	Calf	104,3	4	2	Undetermined	Undetermined	
20-VLT003002	A2020/05745	2020-10-28	Malmö	6141630	1295472	F	Juvenile	118,0	4	1	Undetermined	Undetermined, consistent with drowning but cause undetermined	Bacterial nephritis
20-VLT003026	A2020/05724	2020-07-19	Ängelholm	6242364	1316033	M	Neonate	85,5	3	1	Abandoned	Abandoned	Hepatic and renal lipidosis
20-VLT003027	A2020/05721	2020-07-09	Orust	6533065	1234877	F	Adult	165,0	3	1	Probable bycatch	Probable bycatch	Severe biliary trematode infection
20-VLT003028	A2020/05727	2020-08-09	Kungsbacka	6360713	1243582	M	Neonate	76,0	2	1	Abandoned	Abandoned	Esophageal ulcers; hepatic and renal lipidosis
20-VLT003029	A2020/05720	2020-06-08	Ormestad	6463452	1238894	F	Neonate	71,0	3	3	Abandoned	Abandoned	
20-VLT003030	A2020/05729	2020-08-09	Varberg	6363973	1280853	M	Calf	95,0	3	3	Probable bycatch	Probable bycatch	
20-VLT003031	A2020/05722	2020-07-16	Halmstad	6283323	1314454	M	Juvenile	118,8	2	1	Probable bycatch	Probable bycatch	Mild to moderate liver fibrosis
20-VLT003032	A2020/05726	2020-07-20	Ängelholm	6238380	1314560	F	Adult	154,0	3	3	Trauma	Trauma; uterine rupture	Severe lung parasitism with mild granulomatous pneumonia; moderate to severe biliary trematode infection
20-VLT003140	A2020/05781	2020-07-15	Falkenberg	6304398	1304448	F	Neonate	80,5	2	1	Abandoned	Abandoned	Hepatic lipidosis

[a] Nutritional status: 1=emaciated; 2=poor; 3=normal; 4=robust

[b] Decomposition score: 1= mild; 2=moderate; 3=severe; 4=disintegrating/mummified
